# Supplementary material for: Estimation of the Potentially Avoidable Excess Deaths Associated with Socioeconomic Inequalities in Cancer Survival in Germany
Source: Cancers (Basel). 2021 Jan 19;13(2):357. doi: 10.3390/cancers13020357 (PMC7835812; doi:10.3390/cancers13020357)
Supplement: Supplementary file 1 [file cancers-13-00357-s001.pdf]

cancers

MDPI

Supplementary Material: Estimation of the Potentially Avoidable Excess Deaths Associated With Socioeconomic Inequalities in Cancer Survival in Germany

1. Supplementary Material: Computation of Avoidable Excess Deaths

Here we show an example of how the number of avoidable excess deaths was calculated for the study population and for Germany. We use colorectal cancer as an example. The results differ slightly from the results in the table due to rounding.

The number of observed, expected and excess deaths by deprivation quintile were derived from the case number N and the observed (OS) and expected (ES) survival estimates in the deprivation quintile Q using the following formulas:

$$\text{Number of observed deaths in quintile } Q = N_Q * (1 - OS_Q) \quad (1)$$

$$Number\ of\ expected\ deaths\ in\ quintile\ Q = N_Q * (1 - ES_Q) \quad (2)$$

$$Number\ of\ excess\ deaths\ in\ quintile\ Q = N_Q * \left(ES_Q - OS_Q\right) \quad (3)$$

In quintile 5, absolute and expected survival estimates were 50.5% and 78.6%. Among the 32,029 cases, following the formulas above, the number of observed, expected and excess deaths was 15,854, 6854 and 9000. From the number of excess deaths and the relative survival (RS) estimates in quintile 5 (Table 2: 63.4%) and the relative survival estimate in quintile 1 (Table 2: 67.2%), the number of avoidable excess deaths can be computed using this formula:

$$\begin{aligned} &Number\ of\ avoidable\ premature\ excess\ deaths\ in\ quintile\ Q \\ &= N_Q * ES_Q * \left(RS_{least\ deprived} - RS_Q\right) \end{aligned} \quad (4)$$

For colorectal cancer, 957 excess deaths could be avoided in quintile 5 when the relative survival in quintile 5 was the same as in quintile 1. In the study, we computed these estimates for quintile two to five and summed over the quintiles to obtain the number of avoidable excess deaths and the proportion in relation to all excess deaths, which was 1911 (3.8%) for colorectal cancer (Table 3).

To obtain the number of avoidable excess deaths in Germany, we needed to estimate the number of colorectal cancer patients in each deprivation quintile in Germany. The incidence in Germany was obtained from the database of the Centre for Cancer Registry Data and was 75.7 per 100,000 persons for colorectal cancer (Table 4). From the study population, we estimated the incidence rate ratios for each deprivation quintile (Table 4). By multiplying the incidence rate ratios (e.g., 1.14 for colorectal cancer in Q5) by the total incidence (75.7) we derive the estimated colorectal cancer incidence per quintile (e.g., Q5: 86.5, Table 4). Using the size of the underlying population in Germany per quintile, we get the number of colorectal cancer cases per quintile per year (e.g., Q5 population: 12,229,196 persons). Using these numbers, we estimated that overall, 10,578 colorectal cancer cases were diagnosed per year in municipalities in quintile 5. We now used the survival estimates from above to compute the number of avoidable excess deaths. We get 5236 observed deaths, 2264 expected deaths, 2971 excess deaths and 316 avoidable deaths. In the study, we computed these estimates for quintile two to four and obtained in total 630 avoidable excess deaths (3.9% of all excess deaths, Table 5).

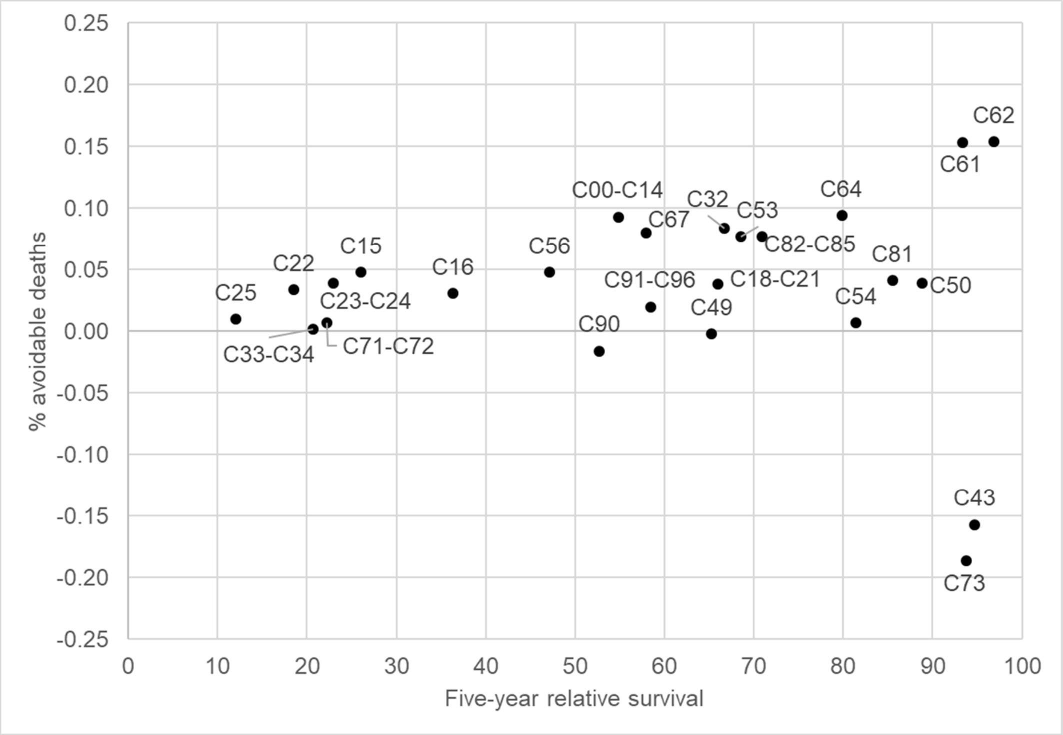

Figure S1. Relationship between the number of avoidable excess deaths in the study population in 2013-17 and relative survival by cancer site.
